# Supplementary material for: Model-based inference of cell cycle dynamics captures alterations of the DNA replication programme
Source: PLoS Comput Biol. 2025 Oct 14;21(10):e1013570. doi: 10.1371/journal.pcbi.1013570 (PMC12543284; doi:10.1371/journal.pcbi.1013570)
Supplement: S1 File — (PDF) [file pcbi.1013570.s001.pdf]

Supporting Information for Model-based inference of cell cycle dynamics captures alterations of the DNA replication programme

Adolfo Alsina

Gulbenkian Institute for Molecular Medicine,

Rua da Quinta Grande 6, 2780-156 Oeiras, Portugal and

GISC, Universidad Rey Juan Carlos,

c. Tulipán, s/n, 28933 Móstoles, Spain

Marco Fumasoni and Pablo Sartori

Gulbenkian Institute for Molecular Medicine,

Rua da Quinta Grande 6, 2780-156 Oeiras, Portugal

Appendix A: Derivation of the likelihood function of measuring a distribution of DNA content in an asynchronous population

In this section we introduce a general likelihood-based approach to infer cell cycle dynamics from high-throughput flow cytometry data. This approach not only takes into account single-cell and population level contributions to the DNA content distribution, but also the impact of technical noise. First, we begin by writing the likelihood of measuring an amount of DNA content y in a given cell as

$$\mathcal{L}(y|\boldsymbol{\theta}) = \int_0^1 P(t) f(y|\boldsymbol{\theta}, t) \mathrm{d}t \,, \tag{A1}$$

where t is the fraction of time elapsed since the start of the cell cycle, $P(t)$ is the age distribution across the population measured in terms of cell cycle progression, $\boldsymbol{\theta} = (\boldsymbol{\theta}_{\text{dyn}}, \boldsymbol{\theta}_{\text{noise}})$ is a parameter vector controlling the cell cycle dynamics and the technical noise and $f(y|\boldsymbol{\theta}, t)$ is the probability of measuring DNA content y given the age of the cell t and the parameters $\boldsymbol{\theta}$.

As the DNA measurements are independent events, the likelihood of observing a certain DNA distribution $\boldsymbol{y} = \{y_1, \ldots, y_N\}$ across the population is given by the product of the individual likelihoods

$$\mathcal{L}(\boldsymbol{y}|\boldsymbol{\theta}) = \prod_{i=1}^{N} \int_0^1 P(t) f(y_i|\boldsymbol{\theta}, t) \mathrm{d}t \,, \tag{A2}$$

where N is the number of cells in the population.

In our model, cell cycle dynamics are fully determined by specifying $\boldsymbol{\theta}_{\text{dyn}}$, corresponding to the relative duration of the different cell cycle phases and the dynamics of DNA content during DNA replication. Therefore, we represent the dynamics by the parameters $\boldsymbol{\theta}^*$ that maximise the likelihood function

$$\boldsymbol{\theta}^* = \text{argmax}_{\boldsymbol{\theta}} \log \mathcal{L}(\boldsymbol{y}|\boldsymbol{\theta}) \,, \tag{A3}$$

where for convenience we maximise the logarithm of the likelihood function. The maximum likelihood estimator (MLE) is the parameter combination that maximises the probability of observing the empirical data given the model.

To obtain a closed expression for the likelihood function (Equation A2) we have to specify $P(t)$, the age distribution across the population, and $f(y|\boldsymbol{\theta}, t)$, the dynamics of DNA content along the cell cycle. First, we focus on the dynamics of DNA content along the cell cycle.

We assumme that every cell follows an identical trajectory since the time of birth ($t = 0$) until the time of division ($t = 1$). Along this trajectory the DNA content of single cells increases monotonically. In particular, DNA content is constant and equal to one copy during G_1 phase, increases deterministically following some monotonically increasing function $f_{\text{det}}(t)$ during S phase, and then remains constant at two copies until the time of division. Additionally, the measured amount of DNA is affected by noise introduced during the measurement process.

We model the measurement noise as a t-Student distribution with location parameter $\mu = 0$, scale parameter proportional to the intensity of the signal $\tau = \sigma f_{\text{det}}$ ([1–3]) and degrees of freedom parametrized by ν. The robustness of the t-Student distribution against the presence of outliers in the data ([4]) makes it a convenient choice to minimise the impact of outliers that are not removed by the preprocessing steps. Taken together, the probability of measuring DNA content y given parameters $\boldsymbol{\theta}$ and the cell age t reads

$$f(y|\boldsymbol{\theta}, t) = \text{Student}(\mu = f_{\text{det}}(t|\boldsymbol{\theta}_{\text{dyn}}), \tau, \nu)\,, \tag{A4}$$

where f_{det} represents the deterministic DNA replication dynamics.

At the population level, we consider populations in exponential growth. In that case, the distribution of cell cycle age across the population also follows an exponential form ([5])

$$P(t) = \log(2)\, 2^{1-t}\,. \tag{A5}$$

Taken together, the model is defined by Equation A2 together with Equation A4 and Equation A5. The maximisation of the log-likelihood function $\log \mathcal{L}$ with respect to the parameters $\boldsymbol{\theta}$ is implemented using CmdStanR 2.32.2 in R 4.4.1.

To quantify goodness of fit, we define a fitting score as $s = 1 - \langle D \rangle$, where $D = \sup_x |F_{\text{data}}(x) - F_{\text{fit}}(x)|$ is the two-sample Kolmogorov-Smirnov statistic quantifying the distance between the empirical and inferred CDFs and the average is taken over 100 samples of size N. Unless specified otherwise, we choose $s^* = 0.95$ as a lower threshold for goodness of fit.

Throughout this manuscript we consider cell populations to be homogeneous and described by a parameter vector $\boldsymbol{\theta}_{\text{dyn}}$. Any differences between cells are attributed to technical noise or their specific positions along the cell cycle. For the sake of completeness, here we briefly describe how to analyse populations where the dynamical parameters $\boldsymbol{\theta}_{\text{dyn}}$ vary across the population.

Assumming the parameters to be distributed across the population according to a probability distribution $P(\boldsymbol{\theta}_{\rm dyn})$ the likelihood function reads

$$\mathcal{L}(\boldsymbol{y}) = \prod_{i=1}^{N} \int_0^1 P(t) P(\boldsymbol{\theta}_{\rm dyn}) f(y_i|\boldsymbol{\theta}, t) \mathrm{d}t \mathrm{d}\boldsymbol{\theta}_{\rm dyn}\,, \tag{A6}$$

where we have assummed that the heterogeneity is only in the dynamical parameters and not in the parameters quantifying the technical noise.

Appendix B: Bayesian analysis of the time fraction distributions

To assess the robustness of the maximum likelihood estimator (MLE), we estimated the posterior parameter distribution using Markov Chain Monte Carlo (MCMC) sampling methods. As this analysis is computationally more intensive than MLE, we focused on the profile shown in Fig 1(d) for illustrative purposes.

Following an initial adaptation period, we sampled a total of 3200 parameter combinations from the posterior distribution using 4 independent chains. As shown in S3 Fig, the marginal parameter distributions are sharply peaked around the MLE (red dashed lines), validating the MLE as a robust estimator of the dynamics. Moreover, the profiles generated from individual parameter combinations show minimal variability compared to the MLE-derived profile, as shown in S4 Fig.

Therefore, we focus on the maximum likelihood estimate (MLE) to characterise the system dynamics throughout this manuscript. However, we note that running a full Bayesian analysis is possible if required.

Appendix C: Benchmarking against existing alternatives

To test the performance of Repliflow, we carried out systematic comparisons against two other widely used approaches: thresholding (TH) and the Dean-Jett-Fox method, as implemented in comercial flow cytometry software. The thresholding approach classifies cells with less than 1.15 DNA content as G_1 and cells with DNA content above 1.8 as G_2/M. On the other hand, as in this manuscript we focus on asynchronous populations, the Dean-Jett-Fox method was implemented following the original Dean-Jett formulation [1].

	**No replication defects**	**Early replication defects**	**Late replication defects**
**Repliflow**	$t_{\mathrm{G_1}} = 20.05$<br>$t_{\mathrm{S}} = 20.61$<br>$t_{\mathrm{G_2/M}} = 59.34$	$t_{\mathrm{G_1}} = 20.64$<br>$t_{\mathrm{S}} = 19.71$<br>$t_{\mathrm{G_2/M}} = 59.65$	$t_{\mathrm{G_1}} = 19.36$<br>$t_{\mathrm{S}} = 20.11$<br>$t_{\mathrm{G_2/M}} = 60.54$
**TH**	$t_{\mathrm{G_1}} = 22.91$<br>$t_{\mathrm{S}} = 13.85$<br>$t_{\mathrm{G_2/M}} = 63.24$	$t_{\mathrm{G_1}} = 25.50$<br>$t_{\mathrm{S}} = 12.16$<br>$t_{\mathrm{G_2/M}} = 62.35$	$t_{\mathrm{G_1}} = 19.75$<br>$t_{\mathrm{S}} = 12.84$<br>$t_{\mathrm{G_2/M}} = 67.41$
**Dean-Jett**	$t_{\mathrm{G_1}} = 21.74$<br>$t_{\mathrm{S}} = 15.39$<br>$t_{\mathrm{G_2/M}} = 62.87$	$t_{\mathrm{G_1}} = 23.13$<br>$t_{\mathrm{S}} = 14.85$<br>$t_{\mathrm{G_2/M}} = 62.02$	$t_{\mathrm{G_1}} = 18.24$<br>$t_{\mathrm{S}} = 24.51$<br>$t_{\mathrm{G_2/M}} = 57.24$

TABLE I. Inferred time fractions for the profiles shown in S5 Fig for the three methods under comparison: Repliflow, thresholding (TH) and Dean-Jett.

We evaluated the performance of the three methods on two independent datasets. First, we generated synthetic DNA profiles for which the fraction of time allocated to each cell cycle phase is exactly known. We generated three profiles with the same time fractions but differing DNA replication dynamics. A visual comparison between the fits produced by Repliflow and Dean-Jett can be seen in S5 Fig. Additionally, we report the inferred fractions using the three methods in Table I

The comparison results show that, despite visually similar fitting results, Repliflow is the only approach that consistently recovers the correct time fractions across all conditions. Repliflow outperforms the alternatives because it is the only approach that explicitly incorporates replication alterations into its model formulation.

To test the performance of the three methods on real data, we turned again to the dataset of [6]. As a summary of our analysis, we report the relative error defined as $\Delta t_i = (t_i - t_{i,0})/t_{i,0}$, where $i \in \{\mathrm{G_1}, \mathrm{G_2/M}\}$, t_i is the fraction of time allocated to phase i inferred by each method and $t_{i,0}$ is the corresponding ground truth. From the full dataset, we filtered 57 profiles where both G1 and G2/M phases could be unambiguously identified. Classification was based on DNA content thresholds: cells with DNA content lower than 1.5 and EdU signal intensity above 10^3 were classified as $\mathrm{G_1}$, while those with DNA content larger than 1.9 copies were classified as $\mathrm{G_2/M}$.The average error across the whole dataset can be found

	**Repliflow**	**Thresholding**	**Dean-Jett**
**G1 (%)**	12.93	21.50	11.10
**S (%)**	17.50	32.11	29.72
**G2/M (%)**	5.29	4.01	18.77

TABLE II. Relative error in the estimation of the fraction of time allocated to G1, S and G2/M across the dataset from Rainey et al., *Cell Reports*, 2020 [6] for the three methods under comparison: Repliflow, thresholding (TH) and Dean-Jett.

in Table II and a graphical representation of the results can be seen in S6 Fig.

The results show that, while specific alternatives might perform similarly to Repliflow for specific phases, Repliflow outperforms them when considering performance across all cell cycle phases simultaneously.

Appendix D: Data processing

1. Gating of raw .fcs files

Raw .fcs files were gated using a custom-made Python script. Gating was automated based on the density of events in the forward (FSC-A) and side (SSC-A) scatter channels by binning and thresholding the data in these channels. In addition, events were also gated and reduced on the DNA dye channel in order to eliminate background noise. Finally, events with intensity lower than 10^4 or larger than $2 \cdot 10^5$ were excluded from further analysis.

The raw .fcs files for the yeast deletion collection were obtained from the files deposited in FlowRepository by [7].

2. Normalisation of the deletion collection results

Our analysis of the deletion collection shows that mutants cluster in two groups in the space of relative phase durations (Fig 2A). In addition to the deletion mutants, the deletion collection dataset contains in-plate controls corresponding to WT strains. While visual inspection of Fig 2A might suggest the presence of two distinct clusters with different mean time fractions, the existence of control profiles in both clusters shows that they are technical

artifacts resulting from batch effects between the different plates.

Therefore, to compare the inference results between different plates, we normalise the fraction of time spent in each cell cycle phase against the in-plate controls. Specifically, we define z-scores for each strain i in phase j as

$$z_{ij} = \frac{\tau_{ij} - \mu_j}{\sigma_j} \,, \tag{D1}$$

where $\tau_{ij} = t_{ij}/t_{j,\mathrm{C}}$ is the ratio between the fraction of time t_{ij} spent by strain i in phase j and the fraction of time $t_{j,\mathrm{C}}$ spent by the in-plate control in phase j, μ_j is the average τ_{ij} across all cells and σ_j is the standard deviation of τ_{ij} across cells.

3. Calculation of the doubling time of cells

RepliFlow infers the relative fraction of time allocated to each cell cycle phase from DNA profiles. To obtain the amount of time that cells spend in each phase we need to independently measure the doubling time of cells T in that particular condition.

To obtain doubling times we measured the growth curves of cells in the required conditions. Specifically, we measured the increase in optical density (OD) every 10 minutes from a dilute regime to saturation in media supplemented with 2% glucose. Maximum growth rates λ were calculated as the largest slope averaged over a sliding window of 9 time points. Doubling times were calculated from the maximum growth rates as $T = \log(2)\,\lambda^{-1}$.

Appendix E: Calculation of the time fractions allocated to each cell cycle phase from cell fractions

Here we describe how to calculate the time fraction t_i allocated to cell cycle phase i from the fraction of cells n_i in phase i estimated via gating. The time fractions are obtained from the cell fractions by inverting the relationship $n_i = \int_{t_i^-}^{t_i^+} \mathrm{d}t P(t)$, where t_i^- (t_i^+) is the fraction of the cell cycle at which phase i begins (ends). Assumming a population in exponential growth, we obtain

$$t_{\mathrm{G}_1} = -\frac{1}{\log(2)} \log\left(1 - \frac{n_{\mathrm{G}_1}}{2}\right) , \tag{E1}$$

$$t_{\mathrm{S}} = -\frac{1}{\log(2)} \log\left(1 - \frac{n_{\mathrm{G}_1} + n_{\mathrm{S}}}{2}\right) - t_{\mathrm{G}_1} \,, \tag{E2}$$

$$t_{\mathrm{G}_2/\mathrm{M}} = 1 - t_{\mathrm{G}_1} - t_{\mathrm{S}} \,. \tag{E3}$$

Appendix F: Detailed derivation of the microscopic model

Here we provide a detailed derivation of the microscopic model presented in the main text. The model describes the coupled dynamics of the fraction of replicated DNA, the number of active forks and the number of licensed origins over time. Although we propose a mean field model where we neglect the spatial structure of the DNA, we account for its impact on the dynamics by introducing effective rates of fork annihilation and passive replication, two processes highly dependent on the spatial configuration of the system.

We begin by deriving an equation for the fraction of replicated DNA. DNA is replicated by replication forks travelling along the genome at a rate that is proportional to the number of active forks at time t

$$\frac{\mathrm{d}}{\mathrm{d}t}\phi = vn\,, \tag{F1}$$

where v is an effective replication velocity. Initially the DNA is fully unreplicated $\phi(t = 0) = 0$.

Next, we focus on the dynamics of the number of active forks. Origins fire with rate γ producing a couple of replication forks as a result. To take into consideration the completion of replicating regions, we assume that forks can collide with a certain rate. As only forks travelling in opposite directions can meet, the rate of collisions is proportional to the relative velocity between the forks times the probability of randomly choosing two forks travelling in opposite directions times two permutations. Together, the collision rate is $2v \cdot \frac{1}{4} \cdot 2 = v$. Moreover, as forks can only travel on unreplicated DNA, we take into account the shortening of the unreplicated region as the dynamics progress and modify the collision rate by introducing a factor proportional to the amount of unreplicated DNA $1 - \phi$. Taken together, the evolution of the number of active forks reads

$$\frac{\mathrm{d}}{\mathrm{d}t}n = 2\gamma\omega - v\frac{n^2}{1-\phi}\,, \tag{F2}$$

where $\omega(t)$ is the number of licensed origins at time t. Additionally, at the start of S phase $n(t = 0) = 0$.

For the number of licensed origins, we account for passive replication of origins by active forks. In this case, the rate is proportional to the relative velocity between the fork and the origin, i.e. the replication fork velocity v. Analogously to the dynamics of the number of active forks, this rate also needs to be modified by a factor proportional to the amount of

unreplicated DNA. Therefore, the dynamics of the number of licensed origins read

$$\frac{\mathrm{d}}{\mathrm{d}t}\omega = -\gamma\omega - v\frac{\omega n}{1-\phi}\,, \tag{F3}$$

with the initial condition $\omega(t=0) = \Omega_0$, where Ω_0 is the total number of identified budding yeast origins.

The above equations together with the initial conditions fully determine the microscopic model.

To generate the synthetic sequencing time course of Fig 5E we run simulations of DNA replication using the inferred microscopic parameters (v, γ) together with the confirmed locations of origins in chromosome V of budding yeast (obtained from [8]). The only source of stochasticity in our simulations are the origin firing times, distributed according to an exponential distribution of parameter γ. The copy number profile $n(x,t)$, characterising the copy number at location x and time t, takes value 1 for unreplicated and 2 for replicated regions. As the replication dynamics is deterministic for each firing time realisation, the profile $n(x,t)$ is uniquely determined given the location and firing times of all origins. To simulate a population of cells, we average the profiles resulting from 100 realisations of the stochastic dynamics.

Appendix G: Obtaining microscopic observables from the DNA sequencing data

Synchronised cell populations are a valuable tool to study cell cycle dynamics. In synchronous experiments cells are synchronised at a particular stage of the cell cycle prior to being released and allowed to cycle freely. By sequencing synchronous populations at different times into S phase, we can get insight into DNA replication dynamics at a fine level of detail.

DNA sequencing experiments of synchronous populations return a time series of average copy number $c_n(x,t)$ for each genomic location x at specific time points. Here, we explain how we extract from this data the dynamical observables required to fit the microscopic DNA replication model: the fraction of replicated DNA, the number of active forks and the number of licensed origins.

First, the fraction of replicated DNA is calculated from the average copy number across

the genome $\bar{c}_n(t)$ as

$$\phi(t) = \bar{c}_n(t) - 1\,, \tag{G1}$$

where $\bar{c}_n(t) = \int dx\, c_n(x,t)$ and the integral is performed over the whole genome.

Next, to calculate the number of active forks, we note that replication origins correspond to peaks in the copy number profiles (Fig 5B). Therefore, we calculate the number of active forks as twice the number of peaks in the smoothed copy number profiles. To reduce the impact of technical noise, we smooth copy number profiles with a Savietsky-Golay filter prior to peak identification and only identify peaks with a prominence of at least five times the noise level.

The number of licensed origins is calculated as the total number of identified budding yeast origins minus the number of passively replicated and fired origins $\omega(t) = \Omega_0 - \omega_\mathrm{p}(t) - \omega_\mathrm{f}(t)$, where $\omega_\mathrm{p}(t)$ is the cumulative number of origins that have been passively replicated up to time t, ω_f is the cumulative number of origins that have fired up to time t and Ω_0=829 is the total number of potential origins in *S. cerevisiae* [8]. ω_f is obtained from the copy number profiles as the cumulative number of distinct peaks. Origins located at position x_0 were considered passively replicated at time t if $c_n(x_0,t) > 1.5$ and x_0 did not correspond to a peak of the copy number profile. The locations of potential origins were obtained from OriDB [8].

Finally, each observable was normalised by its maximum value before fitting to the microscopic model.

Appendix H: Experimental procedures

All strains were derivatives of a modified version (Rad5+) of S. cerevisiae strain W303 (leu2-3,112 trp1-1 can1-100 ura3-1 ade2-1 his3-11,15, RAD5). Growth conditions and flow cytometry analysis were performed as described in [9, 10].

---

[1] P. N. Dean and J. H. Jett, Journal of Cell Biology **60**, 523 (1974).

[2] P. Ubezio and A. Andreoni, Cytometry **6**, 109 (1985).

[3] H. B. Steen, Cytometry **13**, 822 (1992).

[4] K. L. Lange, R. J. A. Little, and J. M. G. Taylor, Journal of the American Statistical Association **84**, 881 (1989).

[5] E. O. Powell, Journal of General Microbiology **15**, 492 (1956).

[6] M. D. Rainey, D. Bennett, R. O'Dea, M. E. Zanchetta, M. Voisin, C. Seoighe, and C. Santocanale, Cell Reports **32**, 108096 (2020).

[7] I. Soifer and N. Barkai, Molecular Systems Biology **10**, 761 (2014).

[8] C. C. Siow, S. R. Nieduszynska, C. A. Müller, and C. A. Nieduszynski, Nucleic Acids Research **40**, D682 (2012).

[9] M. Fumasoni and A. W. Murray, PLOS Genetics **17**, e1009875 (2021).

[10] M. Natalino and M. Fumasoni, Molecular Systems Biology 10.1038/s44320-025-00127-z (2025).
