# Supplementary material for: Model-based inference of cell cycle dynamics captures alterations of the DNA replication programme
Source: PLoS Comput Biol. 2025 Oct 14;21(10):e1013570. doi: 10.1371/journal.pcbi.1013570 (PMC12543284; doi:10.1371/journal.pcbi.1013570)
Supplement: Attachment — Submitted filename: Review_PCOMPBIOL-D-25-00798 - (1).docx [file pcbi.1013570.s008.docx]

Review to “Model-based inference of cell cycle dynamics captures alterations of the DNA replication programme”, submitted to PLOS computational biology

In this paper, the authors developed a computational framework RepliFlow to infer cell cycle phase durations and DNA replication dynamics from DNA content data of asynchronous eukaryotic cell populations, by incorporating biophysical assumptions. They also proposed a microscopic model based on fork velocity and origin firing. To verify the advantages of the proposed methods, further analysis is required. The following are detailed comments:

**Major**

1. (General comments) The authors state that the motivation for this study is that current approaches to infer cell cycle dynamics are not grounded on biophysical models and commonly rely on heuristic definitions. To address this issue, the authors developed a new approach that incorporates biophysical assumptions. However, comparison between the proposed method and conventional approaches is missing. Therefore, readers cannot assess how much the proposed method improves upon conventional methods. The authors should include comparison with conventional methods.

2. Results section, “Inference of cell cycle dynamics recapitulates cell cycle time allocation in S. Cerevisiae”.

(1) In your likelihood model (3) - (4), could you explain the derivation or background for the use of your phenomenological model? Is it enough to use just one parameter $\alpha$? Depending on the range of $\alpha$, replication can be divided into three types (linear, early, and late). Can alpha be time-varying rather than a constant?
(2) Page 2, Equation (5): Why is the basis of the exponential distribution 2? Does it mean that the cell population decreases to half at the end of M phase? If so, is there any reason for this?
(3) In this section, the authors concluded that their approach provides a new way of estimating cell cycle dynamics that is fast, robust, and accurate. However, aside from validation via fitting the DNA content profile of an *S. cerevisiae* population, there is no supporting evidence for the claimed speed and robustness. Please provide additional evidence or benchmarking to substantiate these claims.

3. Results section, “Analysis of the haploid yeast deletion collection captures alterations in the cell cycle”.

(1) Page3, right column, paragraph 3,4: The authors estimated $\alpha$, the degree of nonlinearity in the DNA replication model. However, it is still unclear how $\alpha$ affects the time allocation of the cell cycle phases. Please quantitatively provide the time allocations shown in Fig. 2k and 2l, and compare them with those at $\alpha=1$.

4. Results section, “Quantifying graded perturbations of the cell cycle dynamics”.

(1) The authors used their model to perform inferences under various perturbations, represented in Figure 3. Can you incorporate the amount of perturbation into your model to capture the tendency with respect to the variation of perturbation? Or, you may clarify that incorporating dose (e.g., MMS) into the model would require additional parameters that are computationally much more expensive to obtain.
(2) Page 4, right column, line 8, 9: The authors state “for large MMS concentrations the duration of all cell cycle phases is extended.” However, in Fig. 3(c), $T_{G_{1}}$ looks unchanged as MMS concentration increases. To verify whether the $T_{G_{1}}$ increased, a statistical test is required.

5. Results section, “Characterisation of mammalian replication dynamics from DNA content alone”.

(1) Page 5, right column, line 13-16, and Page 14, Supplementary Figure 3: The authors state that the $G_{1}$ phase is “the only phase that can be unambiguously identified using the gating approach across all conditions (Supplementary Figure 3)”. It is unclear why the Supplementary Figure 3 supports the claim that the $G_{1}$ phase can be unambiguously identified. Please explain how to interpret this figure and why the $G_{1}$ phase can be unambiguously identified.
(2) The authors presented the estimation of the fraction of cells in the $G_{1}$ phase, as it is the only phase that can be unambiguously identified using the gating approach. However, Fig. 4b suggests that other phases may still be identified, even if it is ambiguous. To strengthen the robustness of the method, I suggest including results that assess the estimation accuracy for other cell cycle phases as well.

6. Results section, “Microscopic model of the replication dynamics”.

(1) Although the authors mentioned DNA content f can be replaced by the fraction of replicated DNA $\phi$, it is less clear how your microscopic model (6) - (8) is related to the phenomenological model (3) - (5). Do both models give consistent results? How do two models are connected? Can you derive your phenomenological model from the microscopic model?
(2) In Figure 5(b) and (e), the authors compared simulation with the experiment data. Yet, it is not convincing that the model truly matches the experiment data. Are there any quantitative comparisons between them?
(3) Page 7, Fig. 5(b): This figure is confusing. While Eqs. (6)-(8) are ODEs, Fig. 5(b) shows a spatiotemporal pattern. Please provide a detailed explanation of how the figure was generated using Eqs. (6)-(8).

**Minor**

1. Fig. 1(b): This figure is important for helping readers to understand the assumptions in the DNA replication model. However, some aspects are not reader-friendly. First, the font size is too small to see. Please increase the font size. Second, the scale on the horizontal axis is missing. Please add the label $0, t_{G_{1}},t_{G_{1}}+t_{S}$ and $1$ to the horizontal axis. Third, I think $f(t)$ is or $f_{det,S}(t)$. If so, please correct it accordingly.

2. Page 2, left column, line 7-10: I am confused about whether t represents time or age. Please clarify the definition of t. Also, please clarify whether Fig. 1(c) is time vs P(time) or age vs P(age).

3. Fig. 2(a): Please write down the meaning of the color dots in the caption. Do they indicate any special cases?

4. Page 2, right column, line 33-37: Please add the phrase “(see Materials and Methods)” to this sentence, as readers cannot understand how to calculate the time allocation without referring to Materials and Methods, Appendix C.

5. Fig. 4(b) and Supplementary Fig. 3: Please clearly describe the meaning of the colors in the caption and add a color bar to the figures.

6. Fig. 4(c): In many cases, the dots were slightly below the diagonal. It means that the fraction of $G_{1}$ cells estimated by RepliFlow, tends to be slightly lower than that estimated by EdU. Although I agree that this approach approximately reproduced the result by EdU, I wonder whether it is a limitation of the proposed method.

7. Fig. 4(d): Please write what the error bar, dashed line, and shaded region mean. Also, please clearly write the definition of Early S and Late S in the main text.

8. In Figure 5, panel in (b) is labeled as “simulation” and panel in (e) as “experiment”. However, the figure legend describes panel (e) as “simulated dynamics”. Please verify and correct this inconsistency.

9. In your likelihood model, (3) is a continuous function. (It doesn’t need to be called ‘continuous piecewise’), or it might be called a “piecewise-defined function”

10. In page 6, when describing equations (6) - (8), it is better to say “active forks n over time” if you want it to mean the number of active forks instead of time.

11. Page 9, left column, line 34-36: I cannot find two clusters in Fig. 2(a). Please explain what clusters are.

12. Page 9, right column, line 1: Please write the definition of $t_{ij}$ and $t_{ij,C}$.
