# Supplementary material for: Model-based inference of cell cycle dynamics captures alterations of the DNA replication programme
Source: PLoS Comput Biol. 2025 Oct 14;21(10):e1013570. doi: 10.1371/journal.pcbi.1013570 (PMC12543284; doi:10.1371/journal.pcbi.1013570)
Supplement: Attachment — Submitted filename: reply_to_reviewers_final.docx [file pcbi.1013570.s009.docx]

Dear Dr. Chen,

We sincerely thank you and the reviewers for your time and valuable feedback on our manuscript. The reviewers’ comments have helped us strengthen the paper, and we are grateful for the opportunity to submit a revised version.

In the revised version of the text, we have marked all changes for clarity: additions are shown in blue and removals in red. In addition, we provide below a detailed, point-by-point response to each of the comments raised, along with a description of the corresponding changes made in the revised version.

The two main concerns highlighted by the reviewers have been addressed in detail. First, we have included extensive benchmarking against commonly used alternatives (Dean–Jett and thresholding), demonstrating that RepliFlow achieves superior performance across conditions. Second, we have clarified the role of the microscopic model, presenting it as an illustrative example of the modularity of our framework. We have also discussed its scope and limitations more explicitly, both in the main text and in this response document.

We hope that the revised manuscript now meets the journal’s standards and look forward to your favourable consideration.

**Reviewer #1:** This paper presents an interesting mathematical model of flow cytometry cell cycle profiles. It recapitulates previous findings pertaining to cell cycle perturbations of yeast mutants. While the approach can quantify cell cycle alterations including perturbations within S phase specifically, it is not clear that the approach is superior to other analyses of the same data. In that respect, the authors do not present a comparison and analysis of their data relative to other approaches for analyzing flow cytometry data.

We thank the reviewer for highlighting the need of a benchmarking, a point raised also by the other reviewers. To further validate the performance of Repliflow, we have carried out a detailed comparative analysis against two common approaches used for flow cytometry data analysis: (1) thresholding and (2) the Dean-Jett-Fox method (as implemented in standard flow cytometry software like FlowJo). Below, we provide the details of the implementation:

- Thresholding: In this approach, cells with DNA content <1.15 are classified as G1, those >1.8 as G2/M, and the remaining events as S phase.
- Dean-Jett-Fox: Since in this manuscript we only analyse asynchronous populations, we followed the original Dean-Jett formulation. Importantly, Dean-Jett-Fox has additional free parameters (peak positions, independent variances for each peak, a 3-parameter S-phase model) compared to Replifow. Having fewer parameters, RepliFlow reduces the risk of overfitting without compromising performance.

To evaluate the performance of the three methods we considered two independent datasets:

1. Synthetic data: We generated synthetic DNA profiles for which the fraction of time allocated to each cell cycle phase is known exactly. We generated three profiles with the same time fractions for each phase but different replication dynamics: one profile with defects in early replication, one with late replication defects and a profile with normal progression through S phase.
2. Experimental data from Rainey et al.: We also analysed the performance of the three methods on the dataset of Rainey et al. used to generate Figure 4. In this case, we took the time fractions derived from a gating approach as the ground truth for the comparison.

For the Dean-Jett-Fox and thresholding methods, that estimate the fraction of cells in each cell cycle phase, we have estimated the time fractions assuming a population undergoing exponential growth, as done in the case of Repliflow.

First, we present the comparison results for the synthetic data. The three profiles have been generated considering that G1 and S phase last for 20% of the total cell cycle duration and G2/M accounts for the remaining 60%.

**Figure 1: A)** Synthetic DNA profile without DNA replication defects. **B**) Synthetic DNA profile with early DNA replication defects. **C)** Synthetic DNA profile with late DNA replication defects. All profiles correspond to cell populations that spend 20% of time in G1 and S phase and the remaining 60% in G2/M. Dashed lines indicate the thresholds use for classification in the thresholding method.

|  | **No replication defects** | **Early replication defects** | **Late replication defects** |
| --- | --- | --- | --- |
| **Repliflow** | tG1 = 20.05%  tS = 20.61%  tG2/M = 59.34% | tG1 = 20.64%  tS = 19.71%  tG2/M = 59.65% | tG1 = 19.36%  tS = 20.11%  tG2/M = 60.54% |
| **Thresholding** | tG1 = 22.91%  tS = 13.85%  tG2/M = 63.24% | tG1 = 25.50%  tS = 12.16%  tG2/M = 62.35% | tG1 = 19.75%  tS = 12.84%  tG2/M = 67.41% |
| **Dean-Jett-Fox** | tG1 = 21.74%  tS = 15.39%  tG2/M = 62.87% | tG1 = 23.13%  tS = 14.85%  tG2/M = 62.02% | tG1 = 18.24%  tS = 24.51%  tG2/M = 57.24% |

The comparison results show that, despite the visually similar fitting results, Repliflow is the only approach that consistently recovers the correct parameter values across all three different conditions.

Additionally, we performed further comparisons using the dataset from Rainey et al. (used to generate Figure 4 of the main text). To compare the performance of the three approaches, we applied them to the DNA profiles of the Rainey dataset. Given the challenge of reliably identifying cell cycle phases across all experimental conditions, we restricted our analysis to profiles where both G1 and G2 phases could be unambiguously defined. We classified cells with DNA content lower than 1.5 copies and EdU signal intensity larger than 1E3 as G1 and those with DNA content exceeding 1.9 copies as being in G2/M phase. At the end of this document we provide a table with the specifics of the profiles considered for this analysis.

To assess the performance of each method, we calculated the relative error in the inferred phase durations (G1, G2/M), defined as $e_{i}=\left( t_{i}-t_{i,0} \right)/t_{i,0}$, where $t_{i}$ is the fraction of time allocated to phase I inferred by each method and $t_{i,0}$ the corresponding ground truth.

Across the whole dataset we find the average error to be

|  | **Repliflow** | **Thresholding** | **Dean-Jett-Fox** |
| --- | --- | --- | --- |
| **G1** | 12.93% | 21.50% | 11.1% |
| **S** | 17.50% | 32.11% | 29.72% |
| **G2/M** | 5.29% | 4.01% | 18.77% |

The results show that, while alternative approaches might perform similarly to Repliflow for specific phases, Repliflow outperforms them when considering the performance across all cell cycle phases simultaneously.

Specifically, although the Dean-Jett-Fox algorithm produces visually similar results, it systematically underestimates the time fraction allocated to G2/M. Visual inspection of the results also reveals that Dean-Jett-Fox fails to fit correctly the regions close to the G1 and G2/M peaks resulting from DNA replication defects.

On the other hand, the thresholding method's performance was predictable due to its similarity to the gating approach used as reference. However, its reliance on arbitrarily defined cutoffs makes the results sensitive to both the choice of threshold and alterations of the DNA profile.

Finally, we present the relative error for each method across the complete set of profiles used for the comparison.

To conclude we would like to once again highlight the modularity of our approach. Both the microscopic model and the population model at the core of RepliFlow can be altered, which makes the framework adaptable. More generally, part of our contribution is to advance model-based inference, where the emphasis lies in testing and, if necessary, invalidating models. When a model is invalidated, it can simply be replaced by an alternative. We hope the reviewers appreciate that this does not undermine the method; rather, while any model introduces some degree of bias, our framework is designed to make this bias explicit and controllable.

We have included the results of this comparison in the Materials and Methods.

The authors claim that they can infer perturbations of replication dynamics and also infer parameters of replication dynamics in general. I do not agree with these statements. While they can identify changes specifically in early vs late S phase, flow cytometry and their model cannot distinguish between replication origin loss/gain, changes in origin firing times, and changes in fork speed. It certainly cannot determine where in the genome any such changes (or the normal program for that matter) originate from. A delay in late S phase, for example (and as reported for RRM3 KO, similar to previous reports), could be due to loss of some origins, delays in a variety of replication origin subsets, or slowed replication forks, at specific locations or genome-wide.

Overall, while the mathematical models developed here may be of interest to researchers specializing in such approaches, the biological insights obtained with this work are limited.

We sincerely apologize for any confusion in our original manuscript and greatly appreciate the reviewer’s critique, which has helped us clarify the scope and contributions of our work. We have revised the text to better emphasize the significance of our findings while transparently addressing the limitations noted by the reviewer.

We fully agree on the importance of the limitations highlighted by the reviewer. Some of these constraints are inherent to the experimental data itself, rather than specific to RepliFlow. As the reviewer rightly observes, flow cytometry data cannot resolve microscopic details such as the number of the position of licensed origins. Indeed, the information provided by flow cytometry might be degenerate at the microscopic level, meaning that multiple microscopic configurations can give rise to the same population-level DNA profile and, as a result, be characterised by the same value of our phenomenological parameter alpha. Nonetheless, alpha remains an informative parameter for classifying replication dynamics, as demonstrated by our analysis of different perturbations and genetic knockouts. While additional techniques, such as single cell experiments or detailed microscopic models, would be required to resolve finer mechanistic details, alpha provides a simple and practical metric for comparative studies within the limits of flow cytometry data.

That said, our goal is not to position flow cytometry as superior to alternative methods, but to maximize its informational yield within its inherent limits. As demonstrated in our comparative analyses, RepliFlow outperforms existing tools for flow cytometry data, particularly when analysing strains with altered replication dynamics. We propose it as a rigorous approach to analyse this type of data, while readily acknowledging that other techniques may offer advantages for specific questions.

At the same time, we also note that the microscopic model we introduce should be regarded as an illustrative example that showcases the flexibility of our framework. At present, the model only captures the dynamics of wild-type cells with unperturbed replication programmes. We include it primarily to highlight the modularity of our approach: both microscopic and population-level models can be readily adapted or replaced as needed. Looking ahead, applying the framework to more specific biological contexts will require the development and validation of tailored models of DNA replication accounting for specific perturbations. Nonetheless, even within these constraints, RepliFlow is able to infer parameters from wild-type profiles with an accuracy comparable to sequencing-based approaches.

**Reviewer #2:** In this manuscript the authors present a cell cycle based maximum likelihood based model to infer cell cycle distributions and replication dynamics using flow cytometry data solely stained with a DNA dye (i.e., DAPI). I think this approach is a very welcome addition to the cell cycle analysis toolkit. This is especially strong approach to analyze cell cycle distributions over a large set of perturbations, as demonstrated using the genetic knockout library in yeast. I think this manuscript is suitable for publication in PLoS Computational Biology, following addressing my concerns

Concerns

Although I appreciate the technical nature of this journal, I feel that the manuscript should be re-written with a larger audience in mind. Adoption of these methods is critical for their success. Therefore, I like to see two changes:

• The entire manuscript (especially the result section) should be extensively re-written to read less like a technical manual and take the time to explain design decisions for their application so that their target audience which I assume are both wet-lab biologists as well as computational biologist are able to digest this manuscript. The authors can describe the formulas and nitty-gritty details in the methods section of their manuscript.

We have carefully revised the manuscript to incorporate the results of the analysis presented in this document and to enhance its clarity. As an interdisciplinary team of physicists and biologists, we have strived to structure the manuscript to balance accessibility with technical rigor. While we recognize that the more mathematical section where Repliflow is introduced can be challenging, we believe retaining key equations in the main text is essential for methodological transparency. Nevertheless, for the sake of accesibility we have minimized the number of equations in the main text and provided detailed derivations in the Materials and Methods section. The first Results sub-section, while foundational, is largely self-contained. Readers focusing on the biological applications may proceed directly to subsequent sections without compromising understanding.

• Code repositories should be made available with clear instructions on how to run the associated code (i.e., vignette). This might be even included in the manuscript as Supplementary documentation.

We have now made Repliflow available at github.com/al-sina/repliflow. The Github repository includes a notebook with the code and a reference data file (the profile used for Figure 1d) to test the method.

The assumption that even monogenic cell lines are progressing homogenous through the cell cycle is not necessarily true. For instance, using single cell sequencing revealed that cells at similar stages in S-phase can display different number of forks and replication speeds (van den Berg et al., Nature Methods, 2024). More recently, multigenerational time-lapse imaging has revealed that even sister cells are not progressing at similar rates through the cell cycle (Panagopoulos et al., Nature, 2025). I think the authors should at least cite these works and discuss their implications to their work. But I think a deeper exploration of variability parameters on their maximum likelihood estimation will increase the robustness of their method.

We thank the reviewer for pointing us to these references. We have added them to the manuscript.

To assess the robustness of our maximum likelihood estimator (MLE), we performed Bayesian sampling of the posterior parameter distribution. While computationally more intensive than MLE, this analysis provides valuable insights into parameter uncertainty. We focused on the reference profile shown in Figure 1d for illustrative purposes.

Our analysis demonstrates that the maximum likelihood estimator (MLE) produces robust and reliable parameter estimates. The MLE-derived profile shows strong agreement with the average profile generated from posterior parameter sampling, confirming the consistency of our estimation approach. Furthermore, we observe remarkably low variability among individual sampled profiles relative to the MLE profile.

**Figure 2:** Bayesian analysis of the reference profile shown in Fig. 1d of the main text. The solid black line corresponds to the MLE-derived profile. Solid red lines correspond to the profiles associated to 3200 parameter combinations sampled from the posterior distribution.

Additionally, the marginal distributions are sharply peaked around the MLE (red dashed lines in the figure below). Together, these results validate the MLE as an effective and dependable estimation method for our analysis. Therefore, in the manuscript we only focus on MLE, although we highlight that a Bayesian analysis can be run if needed.

**Figure 3:** Marginal distributions of the Repliflow parameters. Red dashed lines indicate the maximum likelihood estimate of the corresponding parameter.

The analysis and figures presented here have been added to the Supplementary Material.

The authors state G1 are easily identifiable form S-phase cells based on the data from the Rainey et al., 2020 paper. However, I do not understand why they cannot use the G2 population from these data as this seems to be as easily identifiable in these data. It is very unclear why Fig. 8/ Suppl.Fig.3 the author opted to select a Cdc7i for a perturbation from Rainey et al., which I had to figure out myself by comparing resemblance between Suppl. Fig. 3 and Fig. 3a,c,j from Rainey et al., 2020. I personally do not see the problem in identifying the G2 population in these data. Therefore, I strongly feel the authors need to show that their model also predicts G2 proportions in a similar way they can predict fraction of G1 populations.

We apologise for the confusion. We have rewritten the corresponding section to clarify our approach and the limitations of gating. What we meant is that G1 is the only phase that can be robustly identified across the whole Rainey dataset. As the thresholds used to define the phases are heuristically defined, modifying them can strongly impact the number of events assigned to each phase, altering the resulting reference values for the time allocated to each phase. To minimise these effects, we have manually selected 57 profiles for which all phases can be easily identified irrespectively of the specific choice of thresholds. The list of profiles can be found at the end of this document. These profiles have been used for the analysis are presented in the reply to the comments of reviewer 1 as part of the comparison between Repliflow and alternatives.

Furthermore, to show that Repliflow correctly captures the fraction of time allocated to each cell cycle phase from DNA content alone, we have reanalysed the complete Rainey dataset using Repliflow. We have refined the algorithm we used for G1 and G2/M peak identification and compared the inferred time fractions using Repliflow against the values obtained by a gating approach (we refer to the reply to Reviewer 1 for details).

Our results show that Repliflow accurately predicts the time fractions associated to the three cell cycle phases that we consider across all profiles from the Rainey dataset with a fitting score s>0.95.

We have added these results to the main text and updated Figure 4 accordingly. Please note that we present the results now in terms of the fraction of time allocated to each cell cycle phase so we have modified the corresponding Materials and Methods section accordingly.

I am not exactly clear what the additional contribution of their microscopic model of replication dynamics is. In my mind, you still require the position of licensed origins, number of forks and position and fraction of replicated genome. Formally, the only thing this exercise adds is a speed and an origin firing rate? Do these speeds and origin firing rates change in some of the yeast knockout strains? Are these in line with published literature? If the authors want to include this microscopic model in their revisions, they need to extend their analysis to other strains (i.e., knockouts) and show its applicability beyond a single condition.

We thank the reviewer for raising this important point. As it stands, the only requirement of our microscopic model is the position of licensed origins. When fitted to flow cytometry data of WT cells with unperturbed DNA replication dynamics, the model estimates microscopic parameters (origin firing rates and fork velocities) that prove consistent with values obtained through sequencing-based methods. While sequencing data provides a valuable independent validation by allowing comparison of derived dynamic quantities such as the number of active forks and the fraction of replicated genome, we emphasize that our method does not rely on these quantities for inference. This agreement with other established techniques reinforces the model's practical utility.

Regarding how these parameters change in mutant strains, these cases indeed present greater complexity, as the replication programme does not need to proceed normally (a key motivation for introducing alpha in our phenomenological model). As Reviewer 1 rightly points out, these alterations might correspond to several effects including the inactivation of certain subsets of origins, an increase heterogeneity in the firing time distribution, … While we recognise that two parameters cannot fully capture the richness of DNA replication, we view this approach as a proof of principle demonstrating how existing flow cytometry data can provide additional biological information without any added extra cost. We refer to the reply to Reviewer 1 for a detailed discussion of the limitations provided by flow cytometry data and the microscopic model.

The legends, especially of the Supplementary Figures, are absent or meager. The authors need to expand these to describe all features present in the Figures.

In the revised manuscript we have expanded the figure legends to improve clarity.

**Reviewer #3:** Review to “Model-based inference of cell cycle dynamics captures alterations of the DNA replication programme”, submitted to PLOS computational biology

In this paper, the authors developed a computational framework RepliFlow to infer cell cycle phase durations and DNA replication dynamics from DNA content data of asynchronous eukaryotic cell populations, by incorporating biophysical assumptions. They also proposed a microscopic model based on fork velocity and origin firing. To verify the advantages of the proposed methods, further analysis is required. The following are detailed comments:

**Major**

1. (General comments) The authors state that the motivation for this study is that current approaches to infer cell cycle dynamics are not grounded on biophysical models and commonly rely on heuristic definitions. To address this issue, the authors developed a new approach that incorporates biophysical assumptions. However, comparison between the proposed method and conventional approaches is missing. Therefore, readers cannot assess how much the proposed method improves upon conventional methods. The authors should include comparison with conventional methods.

We thank the reviewer for their feedback. For a detailed comparison of our approach with existing alternative methods, we kindly point the reviewer to our response to Reviewer 1's comments. There, we present a systematic evaluation of our method against two alternative approaches, thresholding and the Dean-Jett-Fox algorithm, tested on both synthetic datasets and experimental data.

2. Results section, “Inference of cell cycle dynamics recapitulates cell cycle time allocation in S. Cerevisiae”.

(1) In your likelihood model (3) - (4), could you explain the derivation or background for the use of your phenomenological model? Is it enough to use just one parameter $\alpha$? Depending on the range of $\alpha$, replication can be divided into three types (linear, early, and late). Can alpha be time-varying rather than a constant?

In developing our model, we intentionally began with the simplest possible model of DNA replication, assuming constant DNA replication speed. Such a model accurately describes WT-like profiles and does not require any additional parameters to describe DNA replication dynamics beyond the fraction of time allocated to each phase. Next, to also characterise mutant strains, we extended this approach by proposing the minimal single-parameter model capable of capturing replication defects while maintaining computational tractability. This parsimonious modelling strategy provides a robust foundation that can be systematically expanded as needed for more complex phenotypes. We have rewritten the corresponding section of the manuscript to clarify the rationale behind the phenomenological model.

Regarding the following point, our analysis of the deletion collection indicates that while most mutant profiles can be adequately described by a single parameter, certain mutants require a more complex characterisation. Notably, mutants presenting DNA replication stalling often produce distinctive profiles characterized by an intermediate peak between the G1 and G2/M peaks.

Intuitively, the accurate analysis of these cases would require at least two additional parameters: the time at which stalling occurs (accounting for the position of the intermediate peak) and its duration (accounting for the amplitude of the intermediate peak). We are working on an enhanced version of RepliFlow that incorporates these parameters together with experiments in different conditions to validate the accuracy of such an approach.

We acknowledge that our approach is an oversimplification of the DNA replication programme, as biological dynamics are unlikely to be characterised by a single scalar parameter. While one could theoretically propose a time-dependent function to describe these dynamics more accurately, this would add significant complexity and computational challenges. Nevertheless, the modular nature of RepliFlow allows for progressive modifications of the underlying replication model as needed, as discussed in the previous paragraph in regard to stalling.

(2) Page 2, Equation (5): Why is the basis of the exponential distribution 2? Does it mean that the cell population decreases to half at the end of M phase? If so, is there any reason for this?

The exponential arises from the binary nature of cell division, as each cell produces two daughter cells at the end of the cycle. In an exponentially growing population, the cell count scales as $2^{t/\tau}$ (where $\tau$ is the doubling time). As a result, the distribution of ages in the population is also described by a base-2 exponential function. Importantly, this does not imply population halving during mitosis but rather reflects that each dividing cell finishing the cycle produces two newborn cells that enter the cycle in G1. As a result, in the population there are more cells at the beginning of the cycle than at the end. This feature must be taken into account to infer accurate time fractions from flow cytometry measurements.

(3) In this section, the authors concluded that their approach provides a new way of estimating cell cycle dynamics that is fast, robust, and accurate. However, aside from validation via fitting the DNA content profile of an *S. cerevisiae* population, there is no supporting evidence for the claimed speed and robustness. Please provide additional evidence or benchmarking to substantiate these claims.

To illustrate the robustness of our results, we report the results of a Bayesian analysis in the reply to the comments of Reviewer 2. This analysis demonstrates the consistency of our parameter estimates and provides additional statistical confidence in our findings. We now report credible intervals estimated from the Bayesian analysis for the histogram of Figure 1d in the main text.

Regarding the performance of Repliflow, the maximum likelihood fitting process typically takes between 10 and 180 seconds per profile on a regular laptop, depending on the complexity and specific features, allowing users to analyse large datasets without requiring extensive computing resources.

3. Results section, “Analysis of the haploid yeast deletion collection captures alterations in the cell cycle”.

(1) Page3, right column, paragraph 3,4: The authors estimated $\alpha$, the degree of nonlinearity in the DNA replication model. However, it is still unclear how $\alpha$ affects the time allocation of the cell cycle phases. Please quantitatively provide the time allocations shown in Fig. 2k and 2l, and compare them with those at $\alpha=1$.

We have added the inferred time fractions to the corresponding section of the main text and, for the sake of completeness, we also include them here:

| **Mutant** | **Linear model** | **Alpha model** |
| --- | --- | --- |
| RRM3 | tG1 = 1.24%  tS = 24.95%  tG2/M = 73.81% | tG1 = 3.43%  tS = 31.69%  tG2/M = 64.88% |
| RNR4 | tG1 = 28.10%  tS = 30.92%  tG2/M = 40.98% | tG1 = 21.85%  tS = 34.83%  tG2/M = 43.31% |

Furthermore, regarding the relationship between the parameter alpha and the different time fractions, our analysis shows that these variables are uncorrelated, as shown in Supplementary Fig. 1. Specifically, we observe that variations in the parameter alpha are uncorrelated with changes in the z-scores of any time fraction across the deletion collection.

4. Results section, “Quantifying graded perturbations of the cell cycle dynamics”.

(1) The authors used their model to perform inferences under various perturbations, represented in Figure 3. Can you incorporate the amount of perturbation into your model to capture the tendency with respect to the variation of perturbation? Or, you may clarify that incorporating dose (e.g., MMS) into the model would require additional parameters that are computationally much more expensive to obtain.

Indeed, the goal of that section is to demonstrate RepliFlow's ability to quantify graded perturbations in replication dynamics. Within our framework, the perturbation strength (dose) is intrinsically captured by the effective parameter *α*. By analysing the relationship between the values of *α* and the dose, we can systematically evaluate how dose impacts the replication dynamics. This characterisation provides then a foundation for developing microscopic models that reproduce these dose-dependent effects, thereby offering insights into how specific perturbations influence replication at the molecular level. This rationale has now been made explicit in the discussion.

(2) Page 4, right column, line 8, 9: The authors state “for large MMS concentrations the duration of all cell cycle phases is extended.” However, in Fig. 3(c), $T_{G_{1}}$ looks unchanged as MMS concentration increases. To verify whether the $T_{G_{1}}$ increased, a statistical test is required.

We thank the reviewer for bringing this issue to our attention. To quantitatively assess whether the time allocated to each cell cycle phase increases with MMS concentration, we performed two-sided t-tests comparing phase durations between the two lowest and two highest MMS concentrations. The resulting p-values obtained for each phase are as follows:

- tG1 p-value = 0.3077
- tS p-value = 7.524E-4
- tG2/M p-value = 3.102E-4

Considering these findings, we have modified the main text to include the p-values and to clarify that the data provide statistically significant evidence for increased duration of S and G2/M phases, while no such evidence was found for G1 phase extension.

5. Results section, “Characterisation of mammalian replication dynamics from DNA content alone”.

(1) Page 5, right column, line 13-16, and Page 14, Supplementary Figure 3: The authors state that the $G_{1}$ phase is “the only phase that can be unambiguously identified using the gating approach across all conditions (Supplementary Figure 3)”. It is unclear why the Supplementary Figure 3 supports the claim that the $G_{1}$ phase can be unambiguously identified. Please explain how to interpret this figure and why the $G_{1}$ phase can be unambiguously identified.
(2) The authors presented the estimation of the fraction of cells in the $G_{1}$ phase, as it is the only phase that can be unambiguously identified using the gating approach. However, Fig. 4b suggests that other phases may still be identified, even if it is ambiguous. To strengthen the robustness of the method, I suggest including results that assess the estimation accuracy for other cell cycle phases as well.

We apologise for the confusion. We refer to the reply to reviewer 2 where we have addressed these two comments in detail.

6. Results section, “Microscopic model of the replication dynamics”.

(1) Although the authors mentioned DNA content f can be replaced by the fraction of replicated DNA $\phi$, it is less clear how your microscopic model (6) - (8) is related to the phenomenological model (3) - (5). Do both models give consistent results? How do two models are connected? Can you derive your phenomenological model from the microscopic model?

Whereas the phenomenological model describes the dynamics of DNA content in terms of a single effective parameter (alpha), the microscopic model characterises the replication dynamics using two microscopic parameters: origin firing rate and fork velocity. In this model, DNA content changes are determined entirely by these microscopic parameters.

Both approaches produce consistent results, as shown by the inferred time fractions for the profile shown in Fig. 1d for the alpha model and Fig. 5c for the microscopic. The inferred time fractions are:

- Alpha model: tG1 = 9.70%, tS = 23.30%, tG2/M = 67%.
- Microscopic model: tG1 = 7.10%, tS = 23.03%, tG2/M = 69.87%.

As we can see, both approaches produce consistent estimates of the time fractions allocated to the different cell cycle phases. We have added the inferred time fractions to the corresponding section of the main text.

Regarding the connection between the two models, the alpha provides a reduced representation of DNA replication dynamics that condenses the dependence on the two microscopic parameters (origin firing rate and fork velocity) into a single effective parameter. While this approach accurately captures the overall replication dynamics, it does not provide information about the underlying microscopic dynamics.

In specific cases, the phenomenological model can be understood as an approximation of the microscopic model. For instance, linear replication dynamics, which require a constant overall DNA replication rate. As we assume that the microscopic parameters are time independent, this means that for the majority of S phase replication is carried out by a fixed number of forks travelling with constant velocity. Under these conditions, the dynamics of the system reduce to the phenomenological model with alpha=1.

(2) In Figure 5(b) and (e), the authors compared simulation with the experiment data. Yet, it is not convincing that the model truly matches the experiment data. Are there any quantitative comparisons between them?

We apologise for the confusion. Our proposed microscopic model has no spatial structure, and as such, we did not anticipate quantitative agreement with sequencing profiles. The quantitative validation of our model is presented in panel (d), where we demonstrate that the temporal dynamics of key microscopic observables can be accurately reproduced using parameters inferred from flow cytometry data, despite the lack of spatial structure. The purpose of panel (e) is to illustrate that our minimal model captures qualitative features of the experimental profiles. To obtain a quantitative match between the sequencing profiles and the simulations would require incorporating additional information about genomic organisation, which falls beyond the scope of the current framework.

(3) Page 7, Fig. 5(b): This figure is confusing. While Eqs. (6)-(8) are ODEs, Fig. 5(b) shows a spatiotemporal pattern. Please provide a detailed explanation of how the figure was generated using Eqs. (6)-(8).

We apologise for the confusion. While Fig. 5(e) was generated using the replication parameters (origin firing rate and replication fork speed) inferred using the microscopic model the simulation was not done using the microscopic model itself.

The profile in Fig. 5(e) was generated by simulating a chromosome containing N origins placed at fixed locations, which were sourced from the OriDB database, each with firing rate $\gamma$. Assuming independent origin firing, the firing time distribution is given by an exponential distribution of parameter $\gamma$. The DNA is replicated following the rules shown in Fig. 5(a): after firing, origins become inactivated while two replication forks propagate bidirectionally with constant speed v. Replication forks travel the genome until they encounter another fork travelling in the opposite direction. During this time, any origin that is passively replicated is also rendered inactive.

In the simulations, profiles take binary values: 1 for unreplicated genomic regions and 2 for replicated regions. The profile at time t is then uniquely determined by the location and firing times of all origins. The profiles shown in Fig. 5(e) correspond to the average of 100 independent realisations of the dynamics, with each realisation corresponding to a different set of origin firing times drawn from the exponential distribution.

We have expanded the corresponding section of the Materials and Methods to clarify how Figure 5(e) was generated.

**Minor**

1. Fig. 1(b): This figure is important for helping readers to understand the assumptions in the DNA replication model. However, some aspects are not reader-friendly. First, the font size is too small to see. Please increase the font size. Second, the scale on the horizontal axis is missing. Please add the label $0, t_{G_{1}},t_{G_{1}}+t_{S}$ and $1$ to the horizontal axis. Third, I think $f(t)$ is or $f_{det,S}(t)$. If so, please correct it accordingly.

We have modified the figure accordingly.

2. Page 2, left column, line 7-10: I am confused about whether t represents time or age. Please clarify the definition of t. Also, please clarify whether Fig. 1(c) is time vs P(time) or age vs P(age).

We have rewritten the text to explicitly state that in both Equation 1 and Figure 1c t represents cell age, which corresponds to cell cycle progression. We have also modified the figure caption to make this relationship more transparent for readers.

3. Fig. 2(a): Please write down the meaning of the color dots in the caption. Do they indicate any special cases?

The colored dots correspond to the individual profiles presented in panels h-l. We have now added this information to the figure caption.

4. Page 2, right column, line 33-37: Please add the phrase “(see Materials and Methods)” to this sentence, as readers cannot understand how to calculate the time allocation without referring to Materials and Methods, Appendix C.

We have now added a reference to the Materials and Methods section. For the sake of clarity, we would like to note that the inference of time fractions is described in detail in Appendix A, detailing the functioning of RepliFlow. We have also reworked the Materials and Methods to account for the analysis presented in the reply to Reviewer 2. In the revised version of the manuscript, Appendix E is the section that focuses on converting from cell population fractions obtained via gating to time fractions.

5. Fig. 4(b) and Supplementary Fig. 3: Please clearly describe the meaning of the colors in the caption and add a color bar to the figures.

We have modified the figures accordingly.

6. Fig. 4(c): In many cases, the dots were slightly below the diagonal. It means that the fraction of $G_{1}$ cells estimated by RepliFlow, tends to be slightly lower than that estimated by EdU. Although I agree that this approach approximately reproduced the result by EdU, I wonder whether it is a limitation of the proposed method.

We thank the reviewer for pointing this out. We have reanalysed the complete Rainey dataset for the comparison between Repliflow and alternative methods and updated the corresponding figures in the main text.

As part of this analysis, we have refined the peak identification for the Rainey profiles, improving the accuracy of Repliflow’s inferred values as a result. The updated results show that Repliflow accurately reproduces the gating-derived time fractions not only for G1 but also for S and G2/M for most analysed profiles. Furthermore, the updated values do not show an offset between Repliflow’s estimations and those derived via gating. We refer to the reply to Reviewers 1 and 2 for additional details. Please note that now we report the results in terms of the estimated time fractions rather than fraction of cells in the corresponding cell cycle phase.

7. Fig. 4(d): Please write what the error bar, dashed line, and shaded region mean. Also, please clearly write the definition of Early S and Late S in the main text.

We have expanded the figure caption to explain the meaning of the elements mentioned in the reviewer’s comments and added explicit definitions of early and late S phase to the corresponding section of the main text.

8. In Figure 5, panel in (b) is labeled as “simulation” and panel in (e) as “experiment”. However, the figure legend describes panel (e) as “simulated dynamics”. Please verify and correct this inconsistency.

We thank the reviewer for bringing this issue to our attention. The original figure legend was correct, as panel (b) corresponds to the experimental data and panel (e) to the simulated dynamics. We have corrected this typo in the revised manuscript.

9. In your likelihood model, (3) is a continuous function. (It doesn’t need to be called ‘continuous piecewise’), or it might be called a “piecewise-defined function”

We have edited the text before equation 3.

10. In page 6, when describing equations (6) - (8), it is better to say “active forks n over time” if you want it to mean the number of active forks instead of time.

We have edited the text accordingly.

11. Page 9, left column, line 34-36: I cannot find two clusters in Fig. 2(a). Please explain what clusters are.

We have added an explanatory sentence to the corresponding section.

12. Page 9, right column, line 1: Please write the definition of $t_{ij}$ and $t_{ij,C}$.

We have added this definition to the text.

**List of profiles from Rainey et al. used for the method comparison**

| Fig2D_Expt1_sicon -xl_001.csv | Rainey_001 |
| --- | --- |
| Fig2D_Expt1_sietaa -xl_003.csv | Rainey_003 |
| Fig2D_Expt1_sietaa +xl_007.csv | Rainey_004 |
| Fig2D_Expt1_sirif -xl_002.csv | Rainey_005 |
| Fig2D_Expt1_sirif +xl_006.csv | Rainey_006 |
| Fig2D_Expt1_sirif-etaa -xl_004.csv | Rainey_007 |
| Fig2D_Expt1_sirif-etaa +xl_008.csv | Rainey_008 |
| Fig2D_Expt2_sicon -xl_001.csv | Rainey_009 |
| Fig2D_Expt2_sietaa -xl_003.csv | Rainey_011 |
| Fig2D_Expt2_sietaa +xl_007.csv | Rainey_012 |
| Fig2D_Expt2_sirif -xl_002.csv | Rainey_013 |
| Fig2D_Expt2_sirif +xl_006.csv | Rainey_014 |
| Fig2D_Expt2_sirif-etaa -xl_004.csv | Rainey_015 |
| Fig2D_Expt2_sirif-etaa +xl_008.csv | Rainey_016 |
| Fig2D_Expt3_sicon -xl_001.csv | Rainey_017 |
| Fig2D_Expt3_sietaa -xl_003.csv | Rainey_019 |
| Fig2D_Expt3_sietaa +xl_007.csv | Rainey_020 |
| Fig2D_Expt3_sirif -xl_002.csv | Rainey_021 |
| Fig2D_Expt3_sirif +xl_006.csv | Rainey_022 |
| Fig2D_Expt3_sirif-etaa -xl_004.csv | Rainey_023 |
| Fig2D_Expt3_sirif-etaa +xl_008.csv | Rainey_024 |
| Fig2J_Expt1_atr_002.csv | Rainey_025 |
| Fig2J_Expt1_dmso 1_001.csv | Rainey_032 |
| Fig2J_Expt2_atr_002.csv | Rainey_034 |
| Fig2J_Expt2_dmso 1_001.csv | Rainey_041 |
| Fig2J_Expt3_atr_002.csv | Rainey_043 |
| Fig2J_Expt3_dmso 1_001.csv | Rainey_050 |
| Fig3A_Expt1_16h dmso atri 1h_003.csv | Rainey_052 |
| Fig3A_Expt1_16h dmso atri 4h_005.csv | Rainey_053 |
| Fig3A_Expt1_16h dmso chk1i 1h_007.csv | Rainey_054 |
| Fig3A_Expt1_16h dmso chk1i 4h_009.csv | Rainey_055 |
| Fig3A_Expt1_16h dmso_001.csv | Rainey_056 |
| Fig3A_Expt2_16h dmso atri 1h_003.csv | Rainey_062 |
| Fig3A_Expt2_16h dmso atri 4h_005.csv | Rainey_063 |
| Fig3A_Expt2_16h dmso chk1i 1h_007.csv | Rainey_064 |
| Fig3A_Expt2_16h dmso chk1i 4h_009.csv | Rainey_065 |
| Fig3A_Expt2_16h dmso_001.csv | Rainey_066 |
| Fig3A_Expt3_16h dmso atri 1h_003.csv | Rainey_072 |
| Fig3A_Expt3_16h dmso atri 4h_005.csv | Rainey_073 |
| Fig3A_Expt3_16h dmso chk1i 1h_007.csv | Rainey_074 |
| Fig3A_Expt3_16h dmso chk1i 4h_009.csv | Rainey_075 |
| Fig3A_Expt3_16h dmso_001.csv | Rainey_076 |
| Fig6A_Expt1_sietaa dmso 16h_002.csv | Rainey_082 |
| Fig6A_Expt1_sietaa xl413 16h_006.csv | Rainey_083 |
| Fig6A_Expt1_sietaa-topbp1 dmso 16h_004.csv | Rainey_084 |
| Fig6A_Expt1_sineg dmso 16h_001.csv | Rainey_086 |
| Fig6A_Expt1_sitopbp1 dmso 16h_003.csv | Rainey_088 |
| Fig6A_Expt2_sietaa1a dmso 16h_005.csv | Rainey_090 |
| Fig6A_Expt2_sietaa1a xl413 16h_006.csv | Rainey_091 |
| Fig6A_Expt2_sietaa1a-topbp1 dmso 16h_007.csv | Rainey_092 |
| Fig6A_Expt2_sineg dmso 16h_001.csv | Rainey_094 |
| Fig6A_Expt2_sitopbp1 dmso 16h_003.csv | Rainey_096 |
| Fig6A_Expt3_sietaa1a dmso 16h_005.csv | Rainey_098 |
| Fig6A_Expt3_sietaa1a xl413 16h_006.csv | Rainey_099 |
| Fig6A_Expt3_sietaa1a-topbp1 dmso 16h_007.csv | Rainey_100 |
| Fig6A_Expt3_sineg dmso 16h_001.csv | Rainey_102 |
| Fig6A_Expt3_sitopbp1 dmso 16h_003.csv | Rainey_104 |
